# Successful closure of spontaneous esophageal rupture (Boerhaave’s syndrome) by endoscopic ligation with snare loops

**DOI:** 10.1186/s40064-016-2624-4

**Published:** 2016-06-29

**Authors:** Jun Kuwabara, Yuji Watanabe, You Kojima, Naoyuki Higaki, Yoshiou Ikeda, Kouichi Sato, Motohira Yoshida, Yuji Yamamoto, Satoshi Kikuchi

**Affiliations:** Gastroenterology and Surgical Oncology, Ehime University School of Medicine, Shitsukawa, Toon City, Ehime 791-0295 Japan; Departments of Gastroenterology and Metabology, Ehime University Graduate School of Medicine, Toon City, Japan

**Keywords:** Spontaneous esophageal rupture, Snare, Conservative therapy

## Abstract

**Introduction:**

Spontaneous esophageal rupture is a rare condition with a high mortality rate, and it is generally treated by surgery. In the present report, successful non-surgical closure of spontaneous esophageal rupture by endoscopic ligation with snare loops in a patient with pyopneumothorax and septicemia is presented.

**Case description:**

The case of an 80-year-old man patient with spontaneous esophageal rupture who was cured by endoscopic ligation with snare loops is reported. The patient was admitted with severe chest pain. Chest CT scan revealed pneumomediastinum, and an upper gastrointestinal series using gastrografin showed leakage of contrast medium from the lower esophagus. Therefore, a diagnosis of spontaneous esophageal rupture to the thorax was made. Since the family refused surgery, the patient was treated conservatively. Since extensive blood in the stool was noted on day 5, an emergency endoscopic examination was performed. Clipping was performed around the perforation, and the clips were ligated with snare loops. The patient was discharged on day 83 without recurrence.

**Discussion and evaluation:**

We suggest that endoscopic ligation with snare loops should be chosen for elderly people and high-risk cases.

## Background

Endoscopic ligation with snare loops has mainly been used to prevent hemorrhage and perforation in endoscopic polypectomy. However, the closure of spontaneous esophageal rupture (Boerhaave’s syndrome) by endoscopic ligation has not been reported. In the present report, successful non-surgical closure of spontaneous esophageal rupture by endoscopic ligation with snare loops in a patient with pyopneumothorax and septicemia is presented.

## Case report

**Fig. 1:**
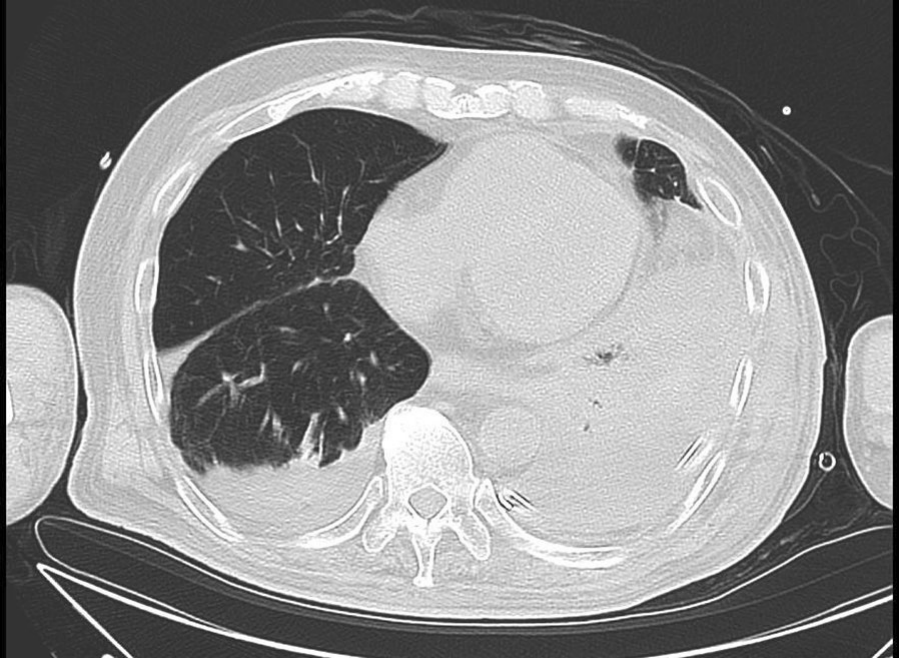
Computed tomography of the chest with pneumohydrothorax and mediastinal air

**Fig. 2:**
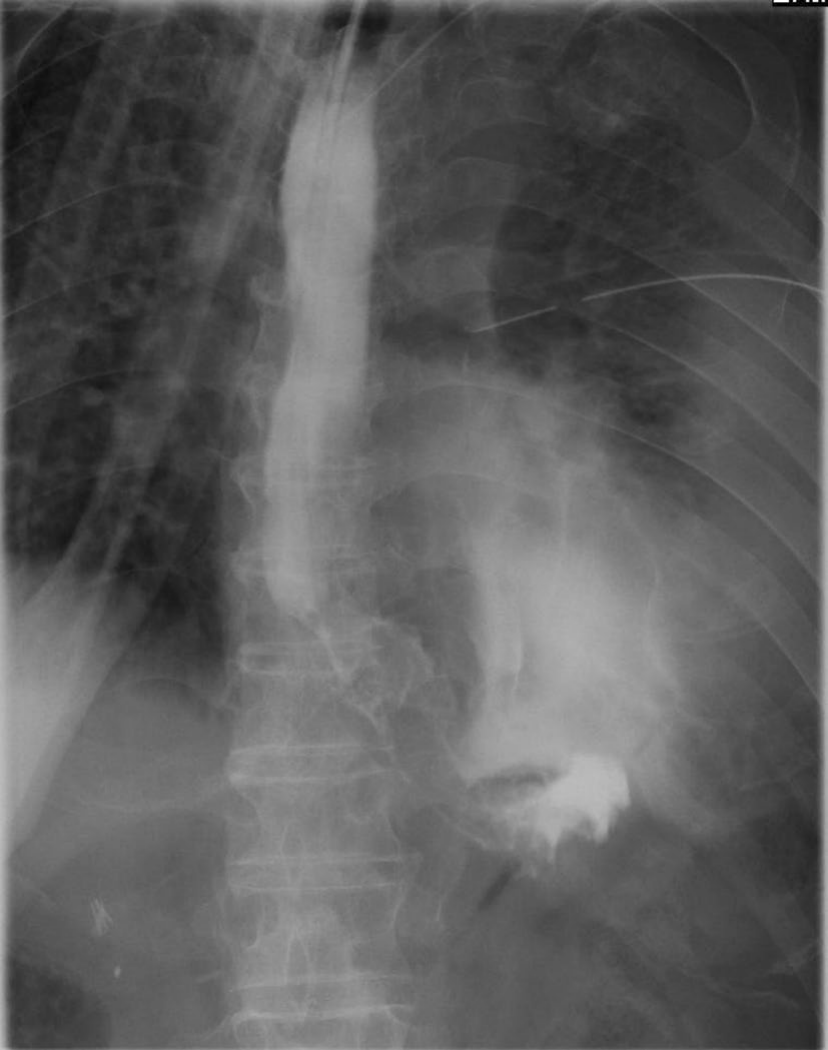
A gastrografin esophagram shows a leak to the left thoracic cavity

**Fig. 3:**
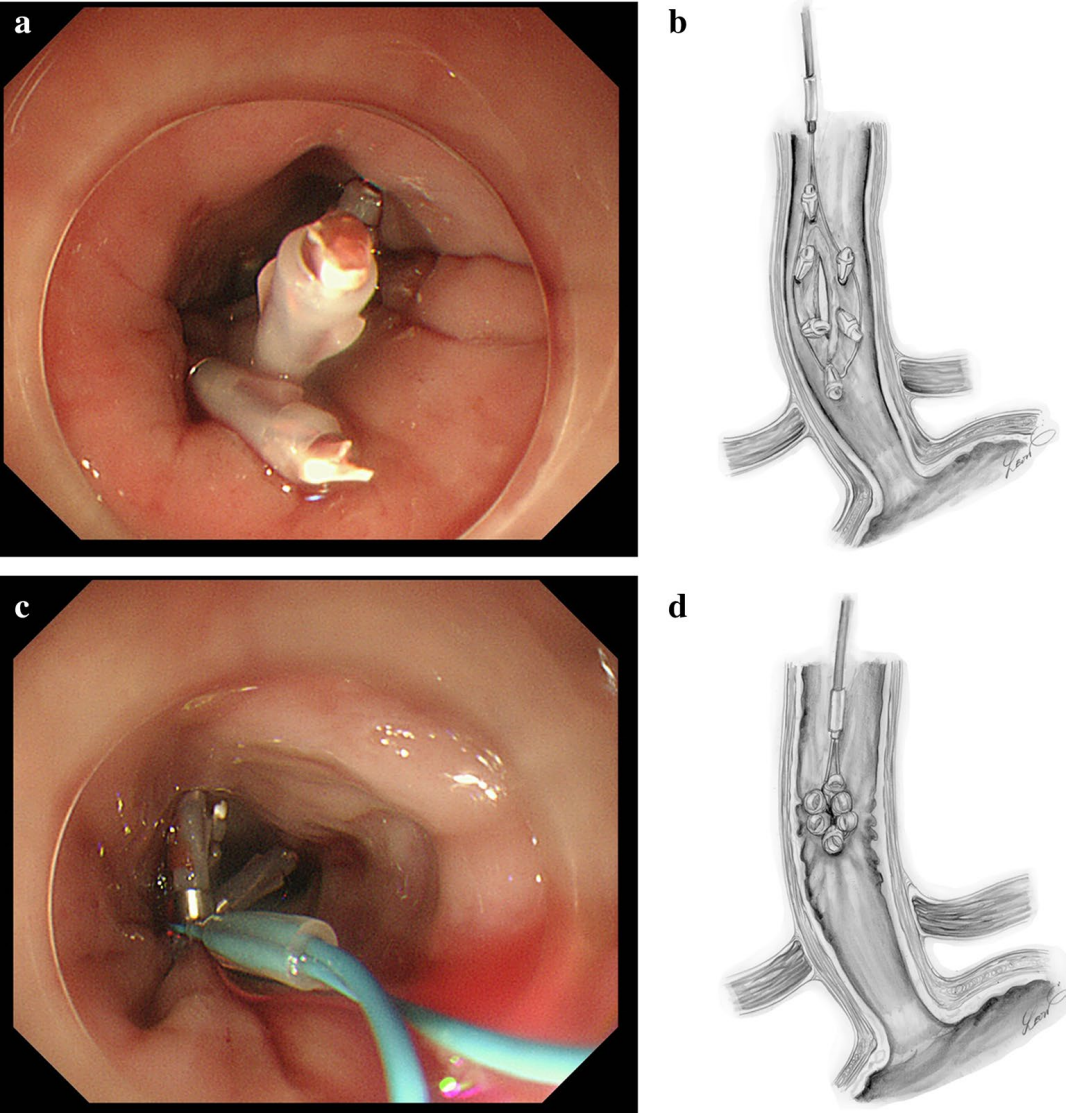
Endoscopic appearance of esophageal perforation before (**a**) and after (**c**) closure using clips with endoscopic ligation with snare loops. Drawings to facilitate understanding of the endoscopic appearance are also shown (**b**, **d**)

**Fig. 4:**
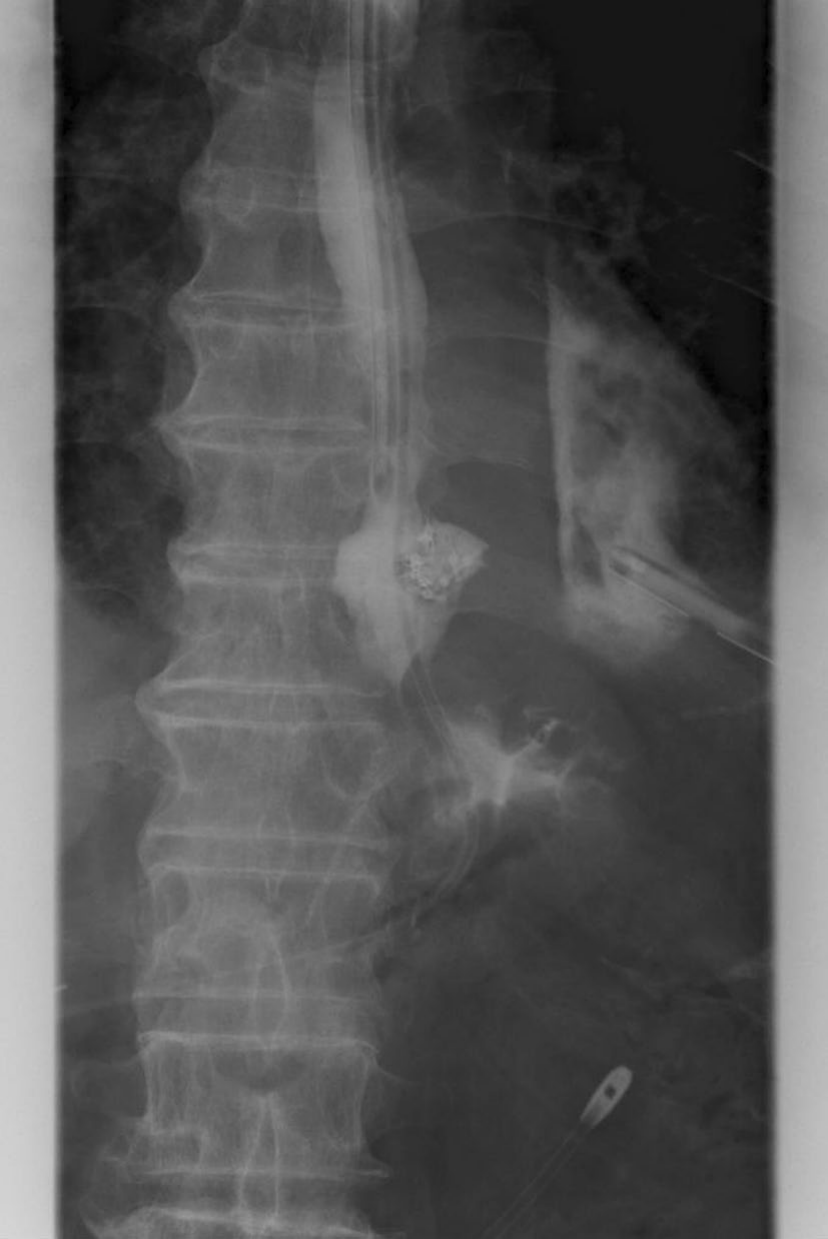
There is no outflow in the thorax on esophagography

**Fig. 5:**
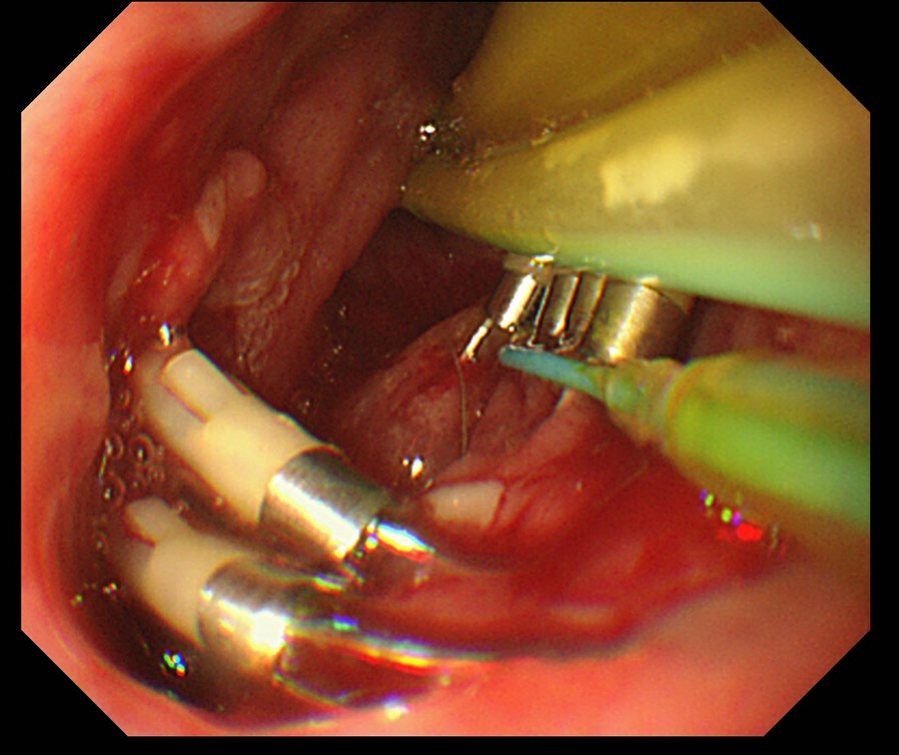
Endoscopic examination shows that the perforation has nearly healed

An 80-year-old man presented to the emergency department complaining of severe chest pain, which began immediately prior to arrival following an episode of vomiting. Physical examination revealed absence of breath sounds over the left hemithorax. Computed tomography of the chest also demonstrated pneumohydrothorax, as well as mediastinal free air (Fig. 1). Laboratory studies showed reduction of leukocytosis at 1.7 (4.0–9.0) × 10^3^/µl, it showed the potential for severe infections. The electrocardiogram showed tachycardia with a ventricular rate of 120 bpm. A 20-French chest tube was inserted, and it drained liquid food residue. Based on the radiographic findings and clinical manifestations, esophageal rupture was the primary diagnosis. The patient was transferred to our hospital where the diagnosis of Boerhaave’s syndrome was confirmed with a gastrografin esophagram (Fig. 2). Since the family did not agree with surgery, the patient received conservative therapy, including withdrawal of oral intake, administration of antibiotics, insertion of a thoracostomy tube and a gastric tube under radiographic guidance, and parenteral nutrition. Since his blood pressure fell and extensive bleeding in the stool was found on day 5, an emergency endoscopic examination was performed. Neither an exposed blood vessel or clear bleeding was evident. Perforation part was located on the left side of the lower esophagus, the length was about 3 cm, the surrounding mucosa was necrosis-like. An attempt was made to close the esophageal perforation by endoscopic clipping, but it was too large. Therefore, clipping was performed around the perforation (Fig. 3a, b), and the clips were ligated with snare loops (Fig. 3c, d). There was a procedure was carried out in a limited space, but is easier, suturing without less than 10 min were completed. The patient was extubated on day 16, and tube feeding of the duodenum was started on day 18. There was no outflow in the thorax on esophagography on day 48 (Fig. 4). At a later date, recurrence findings of perforation in the endoscopy is not recognized, also because of the color of the mucous membrane of the surface was good (Fig. 5), we determined that the lesion has healed, the patient began to oral intake on day 52. The patient was discharged on day 83 without any recurrence.

## Discussion

Spontaneous esophageal rupture was first described by Herman Boerhaave in 1724, while Barrett reported the first successful surgical repair in 1946 (Boerhaave 1955; Barrett 1946). Despite this long history, Boerhaave’s syndrome remains difficult to diagnose early; it is a rare syndrome, seldom presenting with the classical triad of vomiting, chest pain, and subcutaneous emphysema (Walker et al. 1985). Delay in diagnosis reportedly leads to its high overall mortality, especially in the case of high-risk, less treatment burden is selected (Landen and Nakadi 2002).

Among the diagnostic studies, a radiographic examination of the chest usually shows free mediastinal air and hydro- or pneumothorax. At this time, the diagnosis should be strongly suspected and contrast examination of the esophagus performed immediately to demonstrate the rent. Esophagoscopy has also been used as a diagnostic tool (Callaghan 1972).

The diagnosis is often missed initially due to the infrequent occurrence of this syndrome and to the many conditions that in some way mimic it, such as perforated or bleeding ulcers, acute pancreatitis, acute cholecystitis, myocardial infraction, pulmonary embolism, and spontaneous pneumothorax.

Spontaneous esophageal perforation, when the diagnosis is delayed, general condition is poor, made often can not be carried out only conservative medical treatment. If recognized early, surgery in the form of primary repair or gastroesophageal resection is possible. When delayed, infection precludes surgical treatment (Osamu et al. 2015).

Idiopathic esophageal rupture of prognosis is appropriate drainage is related intimately to whether it is carried out. In particular, the drainage in the mediastinum and in the thoracic cavity is important. A drainage tube is often inserted under the CT guide recently. The tube it’s possible to wash is often also used (Yoshinori et al. 2015).

Endoscopic clipping has been used to close esophageal tears and gastric, duodenal, and colonic perforations (Kaneko et al. 1999; Yoshikane et al. 1999), closure of spontaneous esophageal perforations by ligation with snare loops has not been reported. This technique was used to close a post-EMR perforation in the duodenum (Bruckner et al. 1991). Generally, clipping of perforations after 48 h is difficult because the necrotic edges of the perforation cannot hold the clips adequately.

Other forms of endoscopic treatment include placement of temporary plastic endoprostheses in the management of anastomotic leaks (Pross and Ridwelski 2000). Pross et al. described minimally invasive treatment of iatrogenic esophageal perforation by a combination of thoracoscopic posterior mediastinal drainage and deployment of an esophageal self-expanding metal stent (Tatsuro et al. 1997). Metal stents are difficult to remove, and, hence, plastic endoprostheses are preferred whenever subsequent removal of the stent is contemplated.

It has been reported that, if drainage is good, the perforation sites heal at a rate of 1 cm per month (Samarasena et al. 2012). It is believed that, with this treatment, wound healing has been improved.

In recent years, two new techniques are now available that enlarge the possibilities of defect closure: endoscopic vacuum therapy (EVT), and over-the-scope clip (OTSC). EVT is performed by mounting a polyurethane sponge on a gastric tube and placing it into the leakage. Continuous suction is applied via the tube resulting in effective drainage of the cavity and the induction of wound healing, comparable to the application of vacuum therapy in cutaneous wounds. The overall success rate of EVT in the literature ranges from 84 to 100 %, with a mean of 90 %. OTSCs are loaded on a transparent cap which is mounted on the tip of a standard endoscope. By bringing the edges of the perforation into the cap, by suction or by dedicated devices, such as anchor or twin grasper, the OTSC can be placed to close the perforation. For acute endoscopy associated perforations, the mean success rate is 90 % (range 70–100 %). Only few complications have been reported in both techniques (Menningen et al. 2014).

The new device does not require in our techniques. But, it is important to select the one that best suited from a variety of treatments.

## Conclusions

Endoscopic ligation with snare loops may be useful for non-surgical closure of spontaneous esophageal perforation, even if the diagnosis is delayed. It will be necessary to select therapies suitable for patients.
